# Improving accuracy in the estimation of probable dementia in racially and ethnically diverse groups with penalized regression and transfer learning

**DOI:** 10.1093/aje/kwaf001

**Published:** 2025-01-06

**Authors:** Jung Hyun Kim, M Maria Glymour, Kenneth M Langa, Anja K Leist

**Affiliations:** Department of Social Sciences, University of Luxembourg, Esch-sur-Alzette, Luxembourg; Department of Epidemiology, Boston University, MA, United States; Department of Internal Medicine, School of Medicine, University of Michigan, Ann Arbor, MI, United States; Department of Social Sciences, University of Luxembourg, Esch-sur-Alzette, Luxembourg

**Keywords:** race, ethnicity, probable dementia, machine learning, transfer learning, internal validation

## Abstract

Algorithmic estimations of dementia status are widely used in public health and epidemiologic research, but inadequate algorithm performance across racial/ethnic groups has been a barrier. We present improvements in the accuracy of group-specific “probable dementia” estimation using a transfer learning approach. Transfer learning involves combining models trained on a large “source” data set with imprecise outcome assessments, alongside models trained on a smaller “target” data set with high-quality outcome assessments. Transfer learning improves model accuracy by leveraging large-source data while refining estimations with smaller, target data. We illustrate with data from the Health and Retirement Study (source data: *n* = 6630) and the Harmonized Cognitive Assessment Protocol (target data: *n* = 2388). Models for dementia status estimation were evaluated through overall accuracy (Brier score), calibration (intercept, slope), and discriminative ability (area under the receiver operating characteristic curve [AUR] and area under the precision-recall curve [AUPRC]). The transfer-learned algorithm showed higher accuracy compared to the best previously reported algorithm among both non-Hispanic Black participants (Brier 0.049 vs 0.061; AUC 0.84 vs 0.81; AUPRC 0.52 vs 0.39) and Hispanic participants (Brier 0.052 vs 0.056; AUC 0.89 vs 0.87; AUPRC 0.61 vs 0.56). Transfer learning can improve dementia status estimation for groups historically underrepresented in research.

**This article is part of a Special Collection on Methods in Social Epidemiology**.

## Introduction

Dementia affected 57 million people worldwide in 2019,^1^ with an estimated prevalence of 10% among individuals 65 years and older in the United States. Dementia-related inequalities are well documented: African Americans experience the highest incidence and prevalence of dementia among racial and ethnic groups, with nearly twice the prevalence compared to non-Hispanic White individuals in the United States.^2^^,^^3^ Non-White individuals are routinely underrepresented in dementia research studies, however.

Large community-based surveys are valuable resources for studying dementia risk factors due to their comprehensive data on demographics, socioeconomic factors, and health. Such surveys typically collect brief cognitive assessments that can be used to estimate dementia status. However, accurately estimating dementia status for non-Hispanic Black and Hispanic adults using the brief cognitive assessments is challenging due to the small share of data collected from these groups.^4^ Improvement of estimation algorithm accuracy for underrepresented racial and ethnic groups is crucial for the advancement of epidemiologic modeling and research on dementia, as well as for understanding potential racial disparities in dementia risk and outcomes.

Recent efforts have been made to address this challenge by developing algorithms that are sensitive to racial and ethnic information using a subset of population-representative data that has received a clinical assessment for dementia.^5^^,^^6^ A study incorporated race and ethnicity information and its interactions with some of the predictors to estimate the status of dementia and set different cutoff values for classification to achieve similar model performance across racial and ethnic groups.^5^ Another study estimated the status of dementia using a longitudinal, latent-variable model of unobserved cognitive ability and observed predictors such as age, race/ethnicity, and education.^6^ These studies offer valuable insight into building dementia estimation models when there is only a single data set for model construction. However, when additional data containing higher-quality, detailed cognitive assessments are available, the accuracy of the dementia status model can be further improved by combining knowledge coming from models trained on the new data with those trained on the old data instead of solely using either the old or the new data.

**Figure 1:**
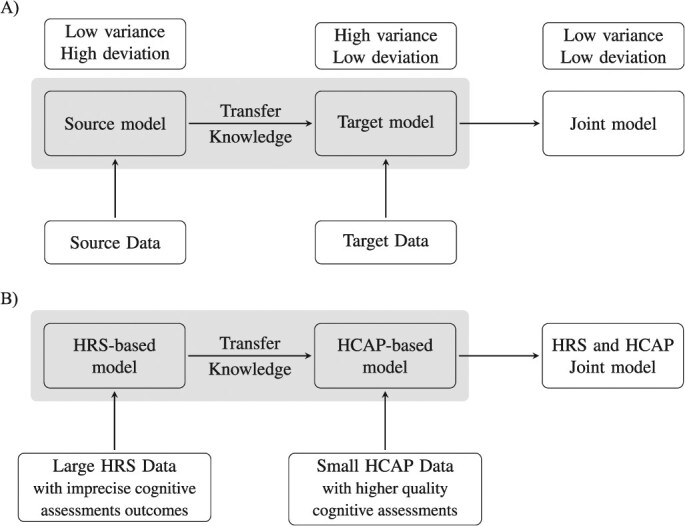
Illustration of transfer learning: (A) conceptual framework and (B) application of the transfer learning method within the context of this study. HCAP, Harmonized Cognitive Assessment Protocol; HRS, Health and Retirement Study.

In this study, our objective is to improve the estimation of dementia status for racially and ethnically diverse groups using a transfer learning with brief cognitive assessments, applicable to large survey data. Transfer learning is a method that leverages large data from a source population with imprecisely measured outcomes to improve estimations for a target population with higher-quality outcome assessments (see Figure 1). The target estimator (ie, the algorithm trained on the target data set) contains high variance due to the small sample size, whereas the source estimator, although more deviated, has low variance. Therefore, jointly using the source and the target data helps solve the deviation-variance trade-off, resulting in improved estimation.^7^^,^^8^ Transfer learning improves the estimation accuracy when there are predictor differences between the target and source data, particularly when these differences have a *sparse structure.*^8^^,^^9^ This means that when comparing the coefficients of the predictors from the 2 models, only a small subset of predictors differs between the 2 data sets. Intuitively speaking, when there are few differences between the source and target predictors, least absolute shrinkage and selection operator (LASSO) regression focuses only on the important differences, resulting in a reduction in the estimation error.

The idea of transfer learning is closely related to the concepts of transportability and generalizability in epidemiologic risk estimation modeling but focuses specifically on improving the measurement of the outcome.^10^^,^^11^ We provide an example case of transfer learning in Appendix S1.

**Figure 2:**
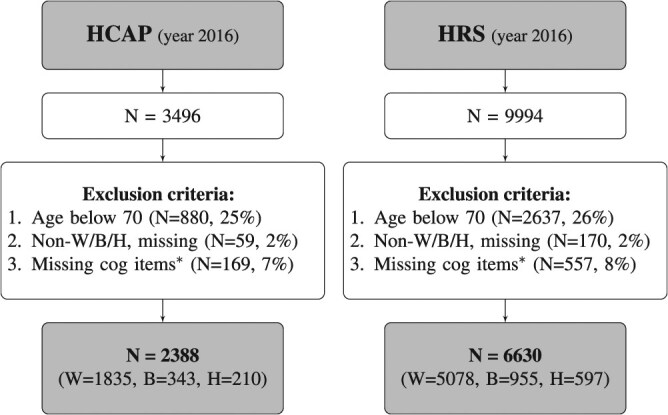
Flowchart of HRS and HCAP samples, summarizing how we arrive at our final sample size. We require criteria 1 to 3 to conform to the existing dementia status estimation algorithms. B, non-Hispanic Black; H, Hispanic; HCAP, Harmonized Cognitive Assessment Protocol; HRS, Health and Retirement Study; missing cog items, missing in cognitive items; W, non-Hispanic White. *We provide the comparative summary of characteristics by this criterion in Tables S1 and  S2.

In our study, we use a large community-based survey as our *source data*, which provide estimated probabilities of dementia using previously introduced algorithms. The source data contain outcomes that are imprecise estimations of dementia status but provide a large sample. Our *target data* are a recently collected subsample with detailed cognitive assessments and dementia classifications but a small sample size. While we might readily construct dementia status estimation models using this new sample, the ongoing challenge is to develop a robust model specifically for Black and Latino participants, who comprise less than 30% of an already small-sized data set. Thus, transporting knowledge of predictor-outcome associations from the large source data might improve the quality of the outcome classifications in the target data despite potential differences between the samples (see Figure 2).

Transfer learning has been used in clinical decision models to mitigate disparities in model performance for small subgroups.^12^ Ultimately, we demonstrate the improvement of dementia status estimation model performance by addressing the deviation-variance trade-off through the joint usage of source and target data.

## Methods

### Data

The Health and Retirement Study (HRS) is a population-based longitudinal study that tracks economic status, family composition, physical health, and cognitive function (randomly assigned either in person or by telephone) of individuals aged 50 years or older living in the United States. The study began in 1992 and has since collected data on more than 43 000 individuals.^13^

The Harmonized Cognitive Assessment Protocol (HCAP) was fielded as a cost-effective method to measure the cognitive function of individuals aged 65 years or older with the aim of facilitating international harmonization.^14^ The HRS-HCAP sample (henceforth HCAP) was a randomly selected subset of the 2016 HRS respondents stratified by household composition. With an eligible sample size of 4425 and a response rate of 80%, the final sample consisted of 3496 individuals; respondents were demographically similar to nonrespondents.^14^

The HCAP sample received an in-depth cognitive function assessment through 1 hour of computer-assisted personal interview that included 5 cognitive domains: memory, executive functioning, language, visuospatial, and orientation. Individuals were assigned to 1 of 3 categories: normal cognition, cognitive impairment, or dementia. The HCAP dementia diagnosis allows the expected scores for cognitive performance on the battery to vary by age, sex, education, race, and ethnicity using a robust normative sample. Therefore, it can classify dementia cases more effectively, potentially identifying cases that might have been overlooked if the participants were compared to distant demographic groups in terms of the characteristics.^3^

We utilized the 2016 HRS interview data for the joint analysis of HRS and HCAP data, including scores of cognitive changes between 2016 and 2014, thus largely following a cross-sectional design. Throughout this article, we used the term *predictor* to describe covariates used as independent variables to estimate and classify prevalent dementia status.

### Participants

Several criteria were applied to the HRS and HCAP data to obtain the final analytical sample (Figure 4). Individuals younger than 70 years were excluded from the model development, as most of the previously developed dementia classification algorithms included only people 70 years and older.^5^^,^^15^ Therefore, we trained the model on data of participants 70 years and older, in line with earlier studies (*n* = 880, 25%). Subsequently, those without race and ethnicity information or who identified themselves as other than non-Hispanic White, non-Hispanic Black, or Hispanic were excluded (*n* = 59, 2%). Individuals without estimated dementia probabilities due to missingness in the cognitive tests were excluded (*n* = 169, 7%). We provide the summary statistics in Table S1 for this criterion. Participants with missing cognitive items were on average older, were more likely to be female, and had a higher degree of functional limitations. The final analytical sample with cognitive status included 2388 participants, of whom 1835 identified as non-Hispanic White, 343 as non-Hispanic Black, and 210 as Hispanic individuals, selected from the HCAP.

Similarly, for the source sample (HRS), individuals younger than 70 years were excluded (*n* = 2637, 26%), as well as those without race and ethnicity information or those who identified themselves as other than non-Hispanic White, non-Hispanic Black, or Hispanic (*n* = 170, 2%). Participants whose estimated dementia probabilities were unknown due to missing data in the cognitive items were excluded (*n* = 557, 8%; see Table S2). The final analytical source sample with cognitive status included 6630 HRS participants, of whom 5078 were identified as non-Hispanic White, 955 as non-Hispanic Black, and 597 as Hispanic individuals.

In cases where individuals were unable to respond directly, due to cognitive impairments or other limitations, a family member, caregiver, or friend was designated to respond on behalf of the individual. The proxy respondent answered questionnaires based on behavioral symptoms to assess cognitive function. Among the final sample of the HCAP data, 6% (*n* = 150) had proxy respondents, and for the HRS data, 8% (*n* = 505) of the final sample comprised proxy respondents.

#### Existing dementia status estimation algorithms

Several dementia status estimation algorithms embedded in the HRS were previously developed based on data from the Aging, Demographics, and Memory Study (ADAMS). ADAMS was a substudy of the HRS that included detailed in-person clinical cognitive assessments of 856 participants 70 years or older. ADAMS was conducted from 2001 to 2008 and established dementia status for each participant.^16^ Algorithms trained on the ADAMS data aimed to estimate the clinical diagnosis of dementia based on a set of predictors available in the main HRS questionnaire, including demographics, a brief cognitive function assessment, activities of daily living, and instrumental activities of daily living.

For proxy respondents, to accommodate the distinct predictor set required, various approaches were used, including the missing indicator method, interaction terms with proxy status, or separate models for proxy respondents. Predictors specifically for the proxy respondents included the 16-item Informant Questionnaire on Cognitive Decline in the Elderly (Jorm IQCODE), a 5-scale proxy-rated memory assessment, and a summary score of Jorm symptoms of cognitive impairment.^4^^,^^17^^,^^18^

For our transfer learning analysis, we used 4 published estimated dementia probabilities accessible on the HRS website: https://hrs.isr.umich.edu/data-products/cognition-data. The 4 dementia probabilities were obtained through algorithms, including the Expert model, a logistic model that uses predictors selected by experts and their interactions; the Lasso model, a model that uses numerous predictors and interacts them with race and ethnicity; the Hurd model, a probit model that uses predictors, including demographic and cognitive items and the changes in cognitive items^5^^,^^15^; and the Latent model, based on a latent-variable model of cognitive function using demographics and cognitive items.^6^ Similar to the dementia diagnosis in the HCAP, the HRS dementia ascertainment algorithms were designed to estimate and classify prevalent dementia status.

#### Predictors used in the transfer learning model

We adopted the predictors used in the original Hurd model.^15^ These predictors included age (categorized in 5-year intervals), education (classified as below 6 years, 6 to 8 years, 9 to 11 years, 12 years, and above 12 years), sex/gender, and cognitive function items, which we provide in detail in Appendix S2. The model also included assessments of limitations in activities of daily living, instrumental activities of daily living, and changes in cognitive item scores during the past 2 waves.

For participants who provided answers through proxy respondents, predictors for measuring cognitive functioning were replaced with IQCODE (see Appendix S2 for further information) and its changes during the past 2 waves. Additionally, the self-response status from the previous survey wave was included. For self-respondents in previous years, cognitive scores from the prior wave were included.

All other listed predictors, except changes in IQCODE and cognitive items from the previous wave, had less than 5% of missingness; we utilized the R package *missForest*^19^ to handle any missing values through random forest imputation. Imputations were performed separately based on the response status by self-report or proxy.

### Statistical analysis

We employed a transfer learning based on the works of Bastani^8^ and Tian and Feng.^9^ Throughout this article, we refer to the HRS with algorithmic dementia probability using the Hurd model as the *source data* and the HCAP with in-depth cognitive status classification as the *target data*.

If the target and source outcome measures are not closely related to each other, then transferring knowledge from the source data might be harmful, which is called negative transfer.^20^ To prevent negative transfer, we evaluated the relevance of the source to the target data using the software package *glmtrans*,^21^ and the results confirmed that this is not a concern. This package uses cross-fold predictions in the target data to assess whether transfer learning improves predictions in a left-out fold.

**Figure 3:**
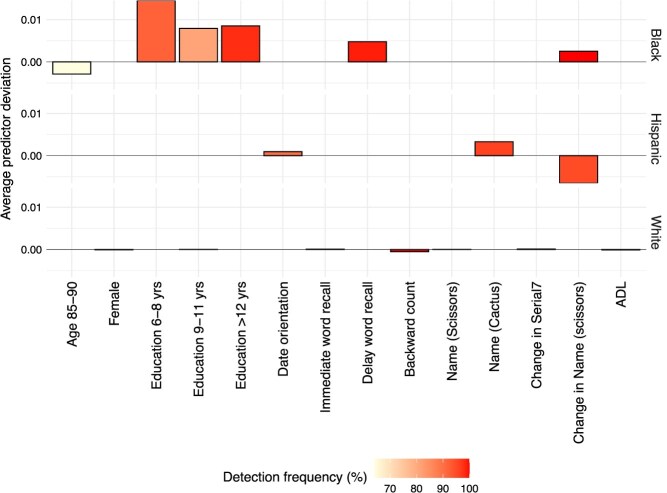
One example of predictor deviation for non-Hispanic Black (top), Hispanic (middle), and White (lower) participants, illustrating the race/ethnicity-specific deviation between the estimator from the HRS (source) data and the estimator from the HCAP (target) data.^8^^,^^9^ Here, we list the deviations that are significant at the 95% level and detected in at least 50% of 1000 runs. ADL, activities of daily living; backward, backward counting; Black, non-Hispanic Black; White, non-Hispanic White; delay, delay word recall; HCAP, Harmonized Cognitive Assessment Protocol; HRS, Health and Retirement Study; IADL, instrumental activities of daily living; immediate, immediate word recall; name(.), name test; Serial7, serial 7 subtraction.

The transfer learning method consists of 2 main steps: gaining knowledge and correcting the deviation.^9^ We provide the graphical abstract of the method in Figure 3. The code for this article is available on the Digital repository: https://github.com/TL-dementia/Code.

#### Step 1: Gaining knowledge from the source data

Risk score algorithms have typically used single data sets to make estimations. Instead of obtaining predictor-outcome associations solely from individual data sets, transfer learning involves the joint use of both source and target data. The first step of the analysis is a standard LASSO regression^22^ to obtain knowledge from the source data, as seen in the following:


(1) \begin{align*} {\hat{\beta}}_{source}\left(\lambda \right)=\mathit{\arg}\underset{\!\!\!\!\!\!\!\!\!\!\!\!\!\!\!\beta }{\min}\left\{{\frac{1}{n_{source}}}\sum_{i=\mathrm{1}}^{n_{source}}\ell\;\Big(\beta; \kern0.24em {\mathbf{X}}^{(i)},{Y}_{source}^{(i)}\Big)+\lambda \parallel \!\beta{\parallel}_1\right\} \end{align*}

Here, ${Y}_{source}$ is the algorithmic dementia probability, $\mathbf{X}$ is the matrices of the predictor set, and ${n}_{source}$ is the number of samples in the source data. We dichotomized ${Y}_{source}$ with an arbitrary threshold value, 0.5, to estimate with a logistic LASSO regression. Experimenting with different thresholds, including 0.25 and 0.75, showed that the performance results were similar to those with a threshold of 0.5, across race and ethnic groups. The term $\lambda \parallel\! \beta{\parallel}_1$ represents the regularization to prevent overfitting, where $\lambda$ controls the strength of the penalty and was chosen to minimize 10 K-fold cross-validation error, and $\parallel\! \beta{\parallel}_1$ is the magnitude of the coefficient vector. The result, denoted as ${\hat{\beta}}_{source}$, is the transferred knowledge, which is the set of coefficients that best fit the LASSO regression model in the HRS. The function $\ell\;\big(\beta; \kern0.24em {\mathbf{X}}^{(i)},{Y}_{source}^{(i)}\big)$ is the standard loss function for a logistic regression for a single sample represented by $\big({\mathbf{X}}^{(i)},{Y}_{source}^{(i)}\big)$. It is obtained through the negative log-likelihood function as follows:


\begin{align*}& \ell\;\big(\beta; \kern0.24em {\mathbf{X}}^{(i)},{Y}_{source}^{(i)}\big)\\&\quad=-\left[{Y}_{source}^{(i)}\log \left(\frac{1}{1+{e}^{-{\mathbf{X}}^{(i)\top}\beta }}\right)+\mathrm{\Big(1}-{Y}_{source}^{(i)}\Big)\log \left(\frac{e^{-{\mathbf{X}}^{(i)\top}\beta }}{1+{e}^{-{\mathbf{X}}^{(i)\top}\beta }}\right)\right] \end{align*}

#### Step 2: Correcting the deviation in the target data

In this second step, our goal is to update the coefficients by minimizing the loss function on the target data. For this reason, our model requires the target and source data to share a core set of common predictors, because the purpose of using the transfer learning method is to fine-tune the source data–derived parameters with the target data. We incorporated a regularization term that penalizes the difference between the coefficients of the target and the source data. The objective function is as follows:


(2) \begin{equation*} {\hat{\beta}}_{TL}\left(\lambda \right)=\mathit{\arg}\underset{\!\!\!\!\!\!\!\!\!\!\!\!\!\!\!\beta }{\min}\left\{{\frac{1}{n_{target}}}\sum_{i=\mathrm{1}}^{n_{target}}\ell\;\big(\beta; \kern0.24em {\mathbf{X}}^{(i)},{Y}_{target}^{(i)}\big)+\lambda \parallel\! \beta -{\hat{\beta}}_{source}{\parallel}_1\right\} \end{equation*}

Here, ${Y}_{target}$ is the detailed cognitive assessment, $\mathbf{X}$ is the matrices of the predictor set, and ${n}_{target}$ is the number of samples in the target data. ${\hat{\beta}}_{source}$ is the coefficient vector from the source data. $\ell\;\big(\beta; \kern0.24em {\mathbf{X}}^{(i)},{Y}_{target}^{(i)}\big)$ is the standard loss function for logistic regression, which remains the same as used in equation (1) but now applied to the target data. The term $\lambda \parallel\! \beta -{\hat{\beta}}_{source}{\parallel}_1$ represents the regularization, where $\lambda$ controls the strength of the penalty and was chosen to minimize 10 K-fold cross-validation error. $\beta -{\hat{\beta}}_{source}$ is the difference between the coefficient for the predictor in the source and target data.^8^ Therefore, when $\lambda$ is close to 1, it sets the coefficients closer to 0 if the deviations between target and source $\beta$ are small. This regularization allows us to select only predictors with larger deviations between the target and the source predictor coefficients. Our final estimate is ${\hat{\beta}}_{TL}$, which is the coefficient vector adjusted to reduce the difference between the target and source data. We guide the readers interested in the proof and more detailed information on this method to the following study.^8^^,^^9^

### Validation and assessment of model performance

Subsequently, we used internal validation with bootstrapping to evaluate the performance of the model. In a simulation study, researchers demonstrated that when the sample size is small, internal validation with bootstrapping is preferred to external validation by keeping the sample complete while correcting for optimism with resampling methods.^23^ Thus, we built a training set by random sampling with replacement from the target data, while the original target data served as a test set. We repeated the entire procedure from the variable selection to model estimation 1000 times and provided an asymptotic standard deviation.

Performances were evaluated by the following metrics^24^^,^^25^: *overall performance* according to the Brier score,^26^  *discriminative ability* by area under the receiver operator characteristic curve (AUC),^27^ area under the precision-recall curve (AUPRC),^28^ and *goodness-of-fit* by calibration slope and intercept.^29^^-^^31^ These measures are briefly summarized in Table 1, and we explain in detail these 3 categories of metrics in Appendix S3.

**Table 1:** Description of the performance measures.

**Category**	**Measure**	**Description**
Overall performance	Brier	Mean squared error between predictions and actual outcome
Calibration	Intercept	Intercept of the predicted probabilities on observed outcome
	Slope	Slope of the predicted probabilities on observed outcome
Discriminative ability	AUC	Aggregated area of a curve between sensitivity and specificity
	AUPRC	Aggregated area of a curve between precision and recall

Abbreviations: AUC, area under the receiver operator characteristic curves; AUPRC, area under the precision-recall curve.

Although overall performance is the summary score, research has suggested that discriminative ability and calibration should be reported in the prediction model as one is not sufficient to represent the different aspects of the model performance.^24^

The method described above was implemented using an open-source statistical software package *glmtrans*^21^ in R version 4.3.0 (R Foundation for Statistical Computing), and Stata version 17.0 (StataCorp LLC) was used for data preparation.

## Results

### Sample characteristics

We compared the characteristics of HCAP participants by dementia status and self-response status in Table 2. HCAP and HRS data have an almost identical distribution of the survey items (Table S3).

**Table 2:** Distributions of the characteristics of the analytical sample of HCAP 2016, stratified by dementia status, racial and ethnic identity, and self/proxy response status.^a^

	**Dementia status**							
	**No dementia**				**Dementia**			
**Proxy status**	**Self-respondents**			**Proxy respondents**	**Self-respondents**			**Proxy respondents**
**Race and ethnicity**	**White**	**Black**	**Hispanic**	**Any race**	**White**	**Black**	**Hispanic**	**Any race**
	***n* = 1578**	***n* = 292**	***n* = 172**	***n* = 53**	***n* = 155**	***n* = 23**	***n* = 18**	***n* = 97**
	**Mean (SD) or %**				**Mean (SD) or %**			
Age, y								
70-74	40%	43%	46%	36%	11%	18%	8.9%	8.7%
75-79	27%	28%	30%	17%	22%	25%	33%	16%
80-84	18%	19%	11%	13%	26%	18%	34%	20%
85-89	8.9%	7.4%	8%	14%	24%	8.6%	24%	21%
$>$89	6.3%	3.8%	4.7%	21%	17%	30%	0%	34%
Female, sex	54%	67%	67%	37%	57%	71%	74%	65%
Schooling, y								
$<$6	0.2%	2.4%	19%	4.7%	1.3%	7%	2.8%	11%
6-8	3.2%	7.8%	24%	18%	3.7%	17%	37%	19%
9-11	9.0%	20%	17%	12%	11%	6.9%	25%	6.4%
12	36%	32%	21%	18%	26%	33%	6.2%	35%
$>$12	34%	37%	19%	40%	32%	51%	29%	28%
Cognitive assessment								
Date orientation	3.72 (0.57)	3.70 (0.57)	3.50 (0.86)		2.82 (1.26)	2.21 (1.37)	2.21 (1.49)	
Immediate recall	5.23 (1.58)	4.58 (1.62)	4.35 (1.91)		3.37 (1.56)	2.66 (1.66)	2.67 (1.66)	
Delayed recall	4.26 (1.87)	3.50 (1.85)	3.43 (2.04)		1.93 (1.76)	1.11 (1.31)	2.00 (1.85)	
Serial 7	3.84 (1.45)	2.40 (1.81)	2.66 (1.92)		2.34 (1.81)	1.54 (1.98)	1.08 (1.14)	
Backward count	95%	86%	86%		83%	73%	76%	
Name (scissors)	99%	98%	97%		95%	96%	98%	
Name (cactus)	98%	82%	91%		80%	42%	77%	
Name (president)	98%	98%	93%		84%	76%	78%	
IQCODE				3.24 (0.39)				4.20 (0.73)
Difficulties in ADL	0.29 (0.80)	0.42 (0.96)	0.59 (1.19)	0.85 (1.57)	0.93 (1.49)	1.57 (1.70)	0.65 (1.52)	3.48 (1.71)
Difficulties in IADL	0.22 (0.64)	0.28 (0.67)	0.60 (1.19)	0.96 (1.49)	1.25 (1.54)	1.71 (1.67)	1.24 (1.71)	4.25 (1.22)

Abbreviations: ADL, activities of daily living; HCAP, Harmonized Cognitive Assessment Protocol; IADL, instrumental activities of daily living; IQCODE, Informant Questionnaire on Cognitive Decline in the Elderly.

a The percentages show the shares of the full sample or the completion rates of the cognitive tests, respectively. Survey weight is applied. Date recall (0-4), immediate recall (0-10), delayed recall (0-10), serial 7 subtraction (0-5), difficulties in ADL/IADL (0-5, higher number worse condition), and IQCODE (0-5, higher number worse condition). The cognitive assessment for proxy respondents was conducted using the IQCODE.

### Model development

For self-respondents, analyses were stratified by race and ethnicity in the source and target data. For proxy respondents, we created a separate model and used the race and ethnicity combined sample with 505 participants from the source data and 150 participants from the target data. The source data were utilized in the transfer learning step to train the model. The model was then debiased using bootstrapped target data, and its performance was assessed using the original target data.

### Model performance

Table 3 compares the performance of the 4 existing models, then re-estimates the Hurd model with the original predictor set using HCAP data (Hurd-HCAP model) and the transfer learning model. For the non-Hispanic Black sample, the Brier score, which measures the overall accuracy, was the best with the transfer learning model compared to the previous 4 models and the Hurd-HCAP model. Additionally, the transfer learning model obtained the calibration intercept closest to zero, meaning that this model contains the least systematic deviation compared to the target estimator.^32^ Discriminative ability was also the highest in the transfer learning model. It is important to note that the performance was evaluated to estimate prevalent dementia, which differs from detecting incident dementia.^33^

**Table 3:** Performance comparisons of various models with HCAP data, using internal validation with bootstrap.^a^

	**Overall accuracy**	**Calibration**		**Discriminative ability**	
	**Brier score**	**Intercept**	**Slope**	**AUC**	**AUPRC**
** Model**	**(Numbers in the subscript are the standard deviations.)**				
Self-respondents					
Black, non-Hispanic					
Hurd	0.064	$-$ 1.23	0.65	0.81	0.46
Expert	0.102	$-$ 1.79	0.48	0.80	0.33
Lasso	0.066	$-$ 1.22	**0.77**	0.82	0.43
Latent	0.070	$-$ 1.58	0.13	0.81	0.37
Hurd-HCAP	0.061 _0.009_	$-$ 1.31 _0.37_	0.29 _0.19_	0.81 _0.05_	0.39 _0.08_
TL	**0.049** _0.003_	$-$ **0.39** _0.29_	0.70 _0.11_	**0.84** _0.02_	**0.52** _0.04_
Hispanic					
Hurd	0.075	$-$ 1.15	0.62	0.86	0.41
Expert	0.091	$-$ 1.27	0.49	0.85	0.35
Lasso	0.088	$-$ 1.35	0.66	0.83	0.41
Latent	0.099	$-$ 1.21	0.19	0.83	0.38
Hurd-HCAP	0.056 _0.015_	$-$ 1.19 _0.34_	0.08 _0.01_	0.87 _0.05_	0.56 _0.10_
TL	**0.052** _0.008_	$-$ **0.07** _0.60_	**0.87** _0.31_	**0.89** _0.03_	**0.61** _0.08_
White, non-Hispanic					
Hurd	0.060	$-$ **0.15**	0.76	0.87	0.50
Expert	0.070	$-$ 0.76	0.60	0.86	0.45
Lasso	0.062	$-$ 0.48	0.81	0.86	0.49
Latent	0.069	$-$ 0.61	0.24	0.85	0.44
Hurd-HCAP	0.057 _0.001_	$-$ 0.21 _0.14_	**0.87** _0.07_	0.87 _0.01_	0.54 _0.01_
TL	**0.056** _0.001_	$-$ 0.20 _0.11_	0.82 _0.04_	**0.88** _0.00_	**0.56** _0.01_
Proxy respondents					
Hurd	0.124	0.15	0.22	0.88	0.91
Expert	0.137	$-$ 0.56	**0.67**	0.88	0.92
Lasso	0.132	$-$ 0.49	0.66	0.88	0.89
Latent	0.156	$-$ 0.21	0.23	0.87	0.92
Hurd-HCAP	0.118 _0.015_	0.10 _0.22_	0.41 _0.24_	**0.90** _0.02_	**0.94** _0.02_
TL	**0.105** _0.008_	$-$ **0.04** _0.22_	0.65 _0.13_	**0.90** _0.01_	0.93 _0.01_

Abbreviations: AUC, area under the receiver operator characteristic curves; AUPRC, area under the precision-recall curve; HCAP, Harmonized Cognitive Assessment Protocol; Hurd-HCAP, Hurd model developed with HCAP data; TL, transfer learning.

a The best model performances are in bold. Brier score: the lower, the better. Calibration intercept: the closer to 0, the better. Calibration slope: the closer to 1, the better. AUC/AUPRC: the higher, the better. By using published dementia probability for Hurd, Expert, Lasso, and Latent models, we do not engage in model development; thus, the standard deviation is not provided.

For Hispanic participants, the calibration intercept and slope largely improved despite large standard deviations using the transfer learning model. Overall, we observed that the accuracy and discriminative ability were moderately better than the existing models.

For the non-Hispanic White sample, the overall accuracy of the transfer learning model was similar to that of the other best-performing existing models. The Hurd-HCAP model outperformed the transfer learning model in terms of calibration, whereas the transfer learning model showed a marginal improvement in discriminative ability.

For proxy respondents, the overall accuracy and the calibration of the transfer learning model were better than the existing models, while the discriminative ability remained almost unchanged. The low-performance gain with the non-Hispanic White sample and proxy respondents reflects the small systematic deviation of the existing algorithms in these groups compared to the target estimator.

### Predictor deviations

We present the predictor deviations between the target and the source estimator in Figure 5 for the case of self-respondents. Here, the deviation refers to the difference between the coefficient for the predictor in the source data (HRS) to the target data (HCAP). Our analysis focused on significant deviations, determined at the 95% confidence level, and was detected more than 500 times out of 1000 runs.^9^ The list of deviations provided is somewhat analysis-specific and may exhibit moderate variations due to the inherent randomness associated with the variable selection process of ${l}_1$ penalized regression, which we employed for deviation detection. However, our findings indicated a consistent pattern in which the magnitude of each deviation tends to be higher for the non-Hispanic Black sample, followed by the Hispanic sample, when compared to the non-Hispanic White sample. We refrain from further stratification by sex/gender due to the limited sample size.

## Discussion

Although algorithmic dementia status estimations are widely used in research, developing algorithms that are valid and reliable for racial and ethnic groups with small samples in existing surveys remains a challenge. Our work aimed to improve the accuracy of group-specific dementia status estimation, with previously reported algorithms as benchmark performance. We employed the transfer learning method, which combines knowledge gained from modeling of large source data with less precise assessment of the outcome and modeling of small target data with more precise assessment of the outcome. An important step in the modeling process is the regularization, which detects and reduces discrepancies in estimated coefficients of the 2 models, similar to the concept of priors or penalties in other modeling strategies.

Transfer learning led to improved performance of the “probable dementia” algorithm in the non-Hispanic Black sample, as indicated by a 20% increase in the Brier score, a 4% increase in the AUC, a 33% increase in the AUPRC, and improved calibration of the model compared to the best previously reported. For the Hispanic sample, we observed a 7% increase in the Brier score, a 2% increase in the AUC, a 9% increase in the AUPRC, and improved calibration of the model compared to the best previously reported.

We built upon the works of previous algorithmic dementia status estimations^5^^,^^6^^,^^15^ and showed further improvement in group-specific performance compared to these models in overall precision, discriminative ability, and calibration. Our adoption of the transfer learning approach aligns with the concepts of transportability and generalizability in epidemiology.^10^^,^^11^ The new method overcomes an important limitation of earlier studies, in which responses to cognitive items were suspected to be differently informative across racial and ethnic groups, by training models on data of different racial and ethnic groups separately.

There are several limitations. First, although we employed the transfer learning method, which effectively used source and target data to mitigate concerns arising from the small sample size, the HCAP data may still be too small to build robust models for each racial and ethnic group.

Second, the model assumed that there were no measurement errors in the diagnosis of HCAP dementia. However, dementia diagnoses in actual clinical settings are based on comprehensive in-person assessments or consensus among multiple experts. In contrast, the HCAP dementia assessment was grounded in a systematic classification that reflects a robust norm sample with similar demographic characteristics. However, an extensive assessment has made misclassification less likely.

Third, our study did not consider the variation within the same racial and ethnic group, which could differ according to factors such as country of birth or skin color, as these aspects are intertwined with experiences of racial discrimination. We also lacked data to develop algorithms for smaller ethnic minority populations, such as Alaskan Native Americans or Asian Americans. A more comprehensive approach would involve a thorough inclusion of variables that fully capture the racialized experience, as we discuss in Appendix S4.

Despite these limitations, this study demonstrated that transfer learning can detect and address deviations between the source and target estimators in existing dementia status estimation models. Our approach combines the advantages of a larger sample in the source data—reducing variance in model parameters due to small samples—with the advantages of higher-quality outcome assessments in the target data—reducing bias in model parameters. Transfer learning improved the estimation performance, particularly for non-Hispanic Black and Hispanic participants, with the transfer-learned algorithm performing better than previously reported algorithms.

Transfer learning is widely applicable in epidemiologic research. For example, self-reported responses, which are simple to collect, may sometimes serve as the source data. The target data could be responses obtained from a subsample of participants that received gold-standard assessments, such as biomarkers from blood or spinal fluid, structural or functional brain measures, or in-depth neuropsychiatric assessments. We can anticipate a more accurate estimation of the disease status by jointly using the target and source data compared to using a single data set. Still, to create a dementia status estimation model that truly represents the population, we require large, high-quality data sets that capture various demographic characteristics of underrepresented groups.

## Supplementary Material

Web_Material_kwaf001

## Data Availability

The HRS data are publicly available at https://hrs.isr.umich.edu/about. The code used in this study is publicly available at https://github.com/TL-dementia/Code.
