# Supplementary material for: Improving accuracy in the estimation of probable dementia in racially and ethnically diverse groups with penalized regression and transfer learning
Source: Am J Epidemiol. 2025 Jan 6;195(1):237–45. doi: 10.1093/aje/kwaf001 (PMC12780771; doi:10.1093/aje/kwaf001)
Supplement: Web_Material_kwaf001 [file web_material_kwaf001.pdf]

Supplementary Material

Improving accuracy in the estimation of probable dementia in racially and ethnically diverse groups with penalized regression and transfer learning

Jung Hyun Kim, M. Maria Glymour, Kenneth M. Langa, Anja K. Leist

Contents

Appendix S1. Example of transfer learning 2

Appendix S2. Detailed explanation of cognitive test items. 2

Appendix S3. Performance metrics. 4

Appendix S4. Race/ethnicity information in dementia status estimation. 5

Table S1. Distributions of characteristics by missing values in cognitive elements, HCAP. 6

Table S2. Distributions of characteristics by missing values in cognitive elements, HRS. 7

Table S3. Distributions of characteristics of analytical sample from HRS 2016 and HCAP. 8

Appendix S1. Example of transfer learning

Small hospitals rely on risk scores that are based on large hospitals' patients. In this case, the target outcome of interest is the small-hospital patients' risk score, and the source outcome is the one based on large-hospital patient cohorts. However, there may be systematic differences in the predictor-outcome associations between source and target data. Although certain predictors might be highly predictive in large-hospital patients, they might be less predictive in small-hospital patients due to differences in physician prescribing patterns or encoding of the medical record, even when there is no true difference in patient characteristics. The transfer learning method first learns the predictor-outcome associations from the large hospital data. Subsequently, to address potential deviation between the estimator built from the large hospital data and that from the small hospital data, the method employs so-called regularization techniques by introducing a penalty term, effectively selecting predictors, and thereby preventing overfitting while minimizing error (for definition see (1, 2)). Transfer learning can be used to effectively capture and minimize the deviation between the source and target estimators while obtaining the minimum error of the final estimator. As a result, the final estimator shows reduced deviation compared to one built from large hospital data and decreased variance compared to the one built from small hospital data. A study found that the highest medical risk performance was achieved by jointly using small and large hospital data rather than using them separately (3).

Appendix S2. Detailed explanation of cognitive test items.

The predictor list follows the original and the modified Hurd dementia estimation model (4, 5).

- *Date recall* is measured by whether the respondent correctly reported today's date (day, week, month, year), and has a score of 0 to 4.
- *Immediate recall* is measured by the number of correctly and immediately recalled nouns and it has score 0 to 10.

- *Delayed recall* is measured by the number of correctly recalled nouns after 5 minutes and has a score of 0 to 10.
- *Serial 7's* is measured by the number of the correct answers of subtracting 7 from 100 consecutively five times and has a score of 0 to 5.
- *Backward count* is measured by the number of correctly counting backward form 10 consecutive numbers from 20. If the respondent successfully performs the task, the score is 1, otherwise 0.
- *Name* is measured by whether the respondent correctly reported the names of the objects (scissors, cactus) and the current president and each has a score of 0 to 1.
- *Changes in cognitive item scores* measures the changes in the scores between the years 2014 and 2016.

Predictors for the proxy respondents included the 16-item *Informant Questionnaire on Cognitive Decline in the Elderly* (Jorm IQCODE). The questions include: 1) Remembering things about family and friends, such as occupations, birthdays, and addresses. 2) Remembering things that have happened recently 3) Recalling conversations a few days later 4) Remembering (her/your) address and telephone number 5) Remembering what day and month it is 6) Remembering where things are usually kept 7) Remembering where to find things which have been put in a different place than usual 8) Knowing how to work familiar machines around the house 9) Learning to use a new gadget or machine around the house 10) Learning new things in general 11) Following a story in a book or on TV 12) Making decisions on everyday matter 13) Handling money for shopping 14) Handling financial matters, that is, (her/your) pension or dealing with the bank 15) Handling other everyday arithmetic problems, such as knowing how much food to buy, knowing how long between visits from family or friends 16) Using (her/your) intelligence to understand what's going on and to reason things through.

If the proxy respondents reported much improvement we gave value 1, a bit improved value 2, same 3, a bit worse 4, much worse 5. We then averaged these responses over 16 items. The final IQCODE had a value between 0 to 5, 5 indicating worse cognitive health

conditions. We operationalized the *change in cognitive status* by the response status. If the respondents' previous wave was answered by IQCODE, we calculated the change in IQCODE score between the two waves. If the respondents' previous wave was self-reported, we included the prior cognitive test score.

Appendix S3. Performance metrics.

The Brier score measures the model's overall accuracy of the performance by the mean squared error between predictions and actual outcomes with scores ranging from 0 to 1, the lower, the better overall accuracy. The calibration slope and intercept show how well the prediction is fitted to the actual observations, and it is obtained from the logistic regression of log odds ratios (In cases where the predicted probability equals zero or one, we replaced the zero to 1×10^{-15} and one to $1 - 1 \times 10^{-15}$.) of the predicted probabilities on observed outcomes with a slope close to 1 and intercept approaching 0 to be the better fit. The calibration intercept measures the systematic bias of the model, and the slope detects overfitting or underfitting (6). AUC is the aggregated area of a curve between sensitivity (true positives/actual positives) and specificity (true negatives/actual negatives) to quantify the discriminative power of the model in all thresholds. The score ranges from 0 to 1, with the higher score, the stronger the discriminative ability. AUPRC is the aggregated area of a curve between precision (true positives/predicted positives) and recall (equivalent to sensitivity, true positives/actual positives) at all thresholds, which is particularly useful when data is highly imbalanced, ranging from 0 to 1 with higher score meaning better discriminative ability (7).

The AUC can be misleading when positive cases are rare. Furthermore, AUPRC can complement by calculating precision and recall with a limited proportion of positive cases. The calibration slope and intercept demonstrate how well the predicted probabilities is fitted to the observed outcomes, which is not captured by either AUC or AUPRC.

Appendix S4. Race/ethnicity information in dementia status estimation.

There are three main approaches to treating the race/ethnicity variable in the dementia status estimation. First, we have the deconstruction of race/ethnicity (8–11). This approach requires a comprehensive inclusion of predictors such as cultural, educational, linguistic background, literacy level (12), early education quality (13), and socioeconomic status (14). Thus, the racialized experience is fully decomposed. This is an ideal approach, as there is no solid evidence of biological differences in race/ethnic groups in dementia. However, it requires high-quality data with maximum inclusion of items that are related to experiences living as racial/ethnic minorities.

The second is to take a race-specific norms or cutoff approach (8, 10), which uses race as a proxy for racialized experiences, and it compares within demographically similar groups to build different cutoffs. However, potential differences in each predictor and why such bias arises remain to be investigated.

Third, the race-specific risk score approach (9, 15). This approach builds a separate prediction model for each racial/ethnic group. It allows to capture race-specific associations of predictors and the outcome, enabling comparisons of predictors across racial/ethnic groups. However, the sample size of racial/ethnic minority groups is often limited for building meaningful separate prediction models.

Table S1. Distributions of characteristics by missing values in cognitive elements, HCAP.

**Characteristic**	**Complete** N = 2388	**Missing** N = 169
	Mean (SD) or %	
**Age, years**		
70-74	26%	17%
75-79	32%	27%
80-84	22%	28%
85-89	12%	20%
90-	7.2%	8.9%
**Female, sex**	59%	66%
**Race/ethnicity**		
Hispanic	8.8%	22%
Unknown	0	1
Black, non-Hispanic	14%	13%
Unknown	0	1
White, non-Hispanic	77%	64%
**Schooling, years**		
<6	3%	12%
6-8	6.3%	9.5%
9-11	12%	14%
12	35%	30%
>12	44%	34%
Unknown	1	0
**Difficulties in ADL**	0.53 (1.18)	0.99 (1.48)
**Difficulties in IADL**	0.53 (1.19)	1.05 (1.54)

Abbreviation. HCAP, Harmonized Cognitive Assessment Protocol; ADL, activities of daily living; IADL, instrumental activities of daily living; SD, standard deviation.

Note. Difficulties in ADL/IADL (0-5, the higher the worse condition).

Table S2. Distributions of characteristics by missing values in cognitive elements, HRS.

Characteristic	Complete N = 6630	Missing N = 557
	Mean (SD) or %	
**Age, years**		
70-74	26%	22%
75-79	32%	28%
80-84	23%	22%
85-89	12%	18%
90-	7.6%	9.3%
**Female, sex**	59%	65%
**Race/ethnicity**		
Hispanic	9.0%	19%
Unknown	0	2
Black, non-Hispanic	14%	15%
Unknown	0	1
White, non-Hispanic	77%	65%
Unknown	0	1
**Schooling, years**		
<6	3.1%	13%
6-8	6.5%	9.4%
9-11	13%	13%
12	35%	30%
>12	43%	35%
Unknown	3	1
**Difficulties in ADL**	0.55 (1.20)	1.04 (1.63)
Unknown	0	9
**Difficulties in IADL**	0.56 (1.23)	1.02 (1.60)
Unknown	0	9

Abbreviation. HRS, Health and Retirement Study; ADL, activities of daily living; IADL, instrumental activities of daily living; SD, standard deviation.
Note. Difficulties in ADL/IADL (0-5, the higher the worse condition).

Table S3. Distributions of characteristics of analytical sample from HRS 2016 and HCAP.

Characteristic	HCAP<br>N = 2388	HRS<br>N = 6630
	Mean (SD) or %	
**Age, years**		
70-74	26%	26%
75-79	32%	32%
80-84	22%	23%
85-89	12%	12%
90-	7.2%	7.6%
**Female, sex**	59%	59%
**Race/ethnicity**		
Hispanic	8.8%	9.0%
Black, non-Hispanic	14%	14%
White, non-Hispanic	77%	77%
**Schooling, years**		
<6	3%	3.1%
6-8	6.3%	6.5%
9-11	12%	13%
12	35%	35%
>12	44%	43%
**Difficulties in ADL**	0.53 (1.18)	0.55 (1.20)
**Difficulties in IADL**	0.53 (1.19)	0.56 (1.23)

Abbreviation. ADL, activities of daily living; HCAP, Harmonized Cognitive Assessment Protocol; HRS, Health and Retirement Study; IADL, instrumental activities of daily living; SD, standard deviation.

Note. Difficulties in ADL/IADL (0-5, the higher the worse condition).

References

(1) Jerome Friedman, Trevor Hastie, and Robert Tibshirani. *The Elements of Statistical Learning*. Vol. 1. Springer Series in Statistics, New York, 2001.

(2) Anja K. Leist et al. “Mapping of machine learning approaches for description, prediction, and causal inference in the social and health sciences”. In: *Science Advances* 8.42 (2022), eabk1942. DOI: 10.1126/sciadv.abk1942.

(3) Hamsa Bastani. “Predicting with Proxies: Transfer Learning in High Dimension”. In: *Management Science* 67.5 (2021), pp. 2964–2984. DOI: 10.1287/mnsc.2020.3729.

(4) Michael D. Hurd et al. “Monetary Costs of Dementia in the United States”. In: *New England Journal of Medicine* 368.14 (2013). PMID: 23550670, pp. 1326–1334. DOI: 10.1056/NEJMsa1204629.

(5) Kan Z Gianattasio, Adam Ciarleglio, and Melinda C Power. “Development of Algorithmic Dementia Ascertainment for Racial/Ethnic Disparities Research in the US Health and Retirement Study”. eng. In: *Epidemiology (Cambridge, Mass.)* 31.1 (Jan. 2020), pp. 126–133. DOI: 10.1097/EDE.0000000000001101.

(6) Ewout W. Steyerberg et al. “Assessing the Performance of Prediction Models: A Framework for Traditional and Novel Measures”. In: *Epidemiology* 21.1 (2010).

(7) Brice Ozenne, Fabien Subtil, and Delphine Maucort-Boulch. “The precision–recall curve overcame the optimism of the receiver operating characteristic curve in rare diseases”. In: *Journal of Clinical Epidemiology* 68.8 (2015), pp. 855–859. DOI: https://doi.org/10.1016/j.jclinepi.2015.02.010.

(8) Jennifer J. Manly. “Advantages and disadvantages of separate norms for African Americans”. In: *The Clinical Neuropsychologist* 19.2 (2005), pp. 270–275. DOI: 10.1080/13854040590945346.

(9) Adam M. Brickman, Raquel Cabo, and Jennifer J. Manly. “Ethical issues in cross-cultural neuropsychology”. In: *Applied Neuropsychology* 13.2 (2006), pp. 91–100. DOI: 10.1207/s15324826an1302_4.

(10) Jennifer J Manly and Ruben J Echemendta. “Race-specific norms: Using the model of hypertension to understand issues of race, culture, and education in Neuropsychology”. In: *Archives of Clinical Neuropsychology* 22.3 (2007), pp. 319–325. DOI: 10.1016/j.acn.2007.01.006.

(11) Jessica P Cerdeña, Marie V Plaisime, and Jennifer Tsai. “From race-based to race-conscious medicine: How anti-racist uprisings call us to act”. In: *The Lancet* 396.10257 (2020), pp. 1125–1128. DOI: 10.1016/s0140-6736(20)32076-6.

(12) Alexander L. Chin, Selamawit Negash, and Roy Hamilton. “Diversity and Disparity in Dementia: The Impact of Ethnoracial Differences in Alzheimer Disease”. In: *Alzheimer Disease & Associated Disorders* 25.3 (2011). DOI: 10.1097/WAD.0b013e318211c6c9.

(13) Shannon Sisco et al. “The Role of Early-Life Educational Quality and Literacy in Explaining Racial Disparities in Cognition in Late Life”. In: *The Journals of Gerontology: Series B* 70.4 (Feb. 2014), pp. 557–567. DOI: 10.1093/geronb/gbt133.

(14) M. Maria Glymour and Jennifer J. Manly. “Lifecourse Social Conditions and Racial and Ethnic Patterns of Cognitive Aging”. In: *Neuropsychology Review* 18.3 (Sept. 2008), pp. 223–254. DOI: 10.1007/s11065-008-9064-z.

(15) Matthew W. Segar et al. “Development and validation of machine learning–based race-specific models to predict 10-year risk of heart failure: A multicohort analysis”. In: *Circulation* 143.24 (2021), pp. 2370–2383. DOI: 10.1161/circulationaha.120.053134.
